# Distinct Luteinization Profiles of Cultured Human Granulosa Cells From Small Antral and Preovulatory Follicles

**DOI:** 10.1210/clinem/dgaf218

**Published:** 2025-04-05

**Authors:** Lea Bejstrup Jensen, Cristina Subiran Adrados, Jane Alrø Bøtkjær, Jesús Cadenas, Sivanandane Sittadjody, Emmanuel Opara, Pernille Landbæk Sørensen, Kirsten Tryde Macklon, Anette Tønnes Pedersen, Stine Gry Kristensen

**Affiliations:** Laboratory of Reproductive Biology, University Hospital of Copenhagen, Rigshospitalet, Copenhagen 2100, Denmark; Laboratory of Reproductive Biology, University Hospital of Copenhagen, Rigshospitalet, Copenhagen 2100, Denmark; The Fertility Clinic, Department of Gynaecology, Fertility and Obstetrics, University Hospital of Copenhagen, Rigshospitalet, Copenhagen 2100, Denmark; Laboratory of Reproductive Biology, University Hospital of Copenhagen, Rigshospitalet, Copenhagen 2100, Denmark; Wake Forest Institute for Regenerative Medicine, Wake Forest School for Medicine, Winston-Salem, NC 27157, USA; Wake Forest Institute for Regenerative Medicine, Wake Forest School for Medicine, Winston-Salem, NC 27157, USA; School of Biomedical Engineering and Sciences (SBES), Wake Forest School of Medicine, Winston-Salem, NC 27157, USA; Laboratory of Reproductive Biology, University Hospital of Copenhagen, Rigshospitalet, Copenhagen 2100, Denmark; The Fertility Clinic, Department of Gynaecology, Fertility and Obstetrics, University Hospital of Copenhagen, Rigshospitalet, Copenhagen 2100, Denmark; The Fertility Clinic, Department of Gynaecology, Fertility and Obstetrics, University Hospital of Copenhagen, Rigshospitalet, Copenhagen 2100, Denmark; Department of Clinical Medicine, University of Copenhagen, Copenhagen 2200, Denmark; Laboratory of Reproductive Biology, University Hospital of Copenhagen, Rigshospitalet, Copenhagen 2100, Denmark

**Keywords:** luteinization, granulosa cells, hormone secretion, small antral follicles, preovulatory follicles, timeline

## Abstract

**Context:**

The transformation of follicular granulosa cells into luteal cells of the corpus luteum remains poorly understood in the human ovary.

**Objective:**

To investigate the luteinization process and steroidogenic differences between granulosa cells from small antral and preovulatory follicles in vitro.

**Methods:**

At the University Hospital of Copenhagen, Denmark, and Wake Forest Institute for Regenerative Medicine, USA, granulosa-lutein cells were obtained from 12 women undergoing IVF treatment, while follicular granulosa cells from unstimulated small antral follicles and corpus luteum were collected from 18 women undergoing ovarian tissue cryopreservation. Cells were cultured for up to 96 hours or 12 days with or without androstenedione or testosterone supplementation and analyzed using RT-qPCR and steroid hormone assays.

**Results:**

In follicular granulosa cells, luteinization markers (CYP11A1, *P* < .05; STAR, *P* < .001) increased within 24 to 48 hours, while granulosa markers (HSD17β1, *P* < .001; CYP19A1, *P* < .05) decreased within 6 to 12 hours. Luteinizing hormone/choriogonadotropin receptor remained unchanged. By 48 hours, gene expression resembled that of the corpus luteum. In contrast, granulosa-lutein cells exhibited highly luteinized profiles from day 0, with significantly higher progesterone/(17)estradiol ratios. Androgen supplementation and long-term follicle-stimulating hormone exposure did not alter luteinization.

**Conclusion:**

This study uniquely demonstrates that unstimulated follicular granulosa cells undergo a gradual, intrinsic luteinization process, independent of external hormonal triggers. In contrast, granulosa-lutein cells are already highly luteinized upon aspiration. These findings challenge conventional views on luteinization and highlight intrinsic cellular programming as a key driver, offering new insights into ovarian physiology and potential therapeutic targets for reproductive disorders.

Granulosa cells are central to female reproductive function, involving key processes in ovarian follicle development, steroidogenesis, and oocyte maturation. Together with theca cells, granulosa cells play a vital role in maintaining the balance of estrogen and progesterone production, which drives both ovarian function and the hypothalamic-pituitary-ovarian axis (1, 2). During folliculogenesis, granulosa cells undergo massive proliferation when transitioning from an undifferentiated state to a differentiated phenotype as the follicle progresses toward ovulation. This differentiation is triggered by a surge in the luteinizing hormone (LH), which initiates luteinization, transforming the granulosa cells into large luteal cells (3). This process is characterized by enhanced luteinizing hormone/choriogonadotropin receptor (LHCGR) expression on the granulosa cells and a shift from primary estradiol to progesterone production (4, 5). This transformation is crucial to establish and maintain the corpus luteum, in which high levels of progesterone are produced to support early pregnancy if fertilization occurs (6-8). While the process of luteinization and progesterone production varies significantly across species, the corpus luteum has been extensively studied. However, the mechanisms underlying luteinization, particularly in humans, are not yet fully elucidated.

Early studies demonstrated that granulosa cells undergo significant structural and functional changes in vitro, transitioning from fibroblast-like cells to a pavement-like epithelial phenotype within 48 hours of culture (9-11). These changes are accompanied by a marked increase in progesterone production, which marks the rapid differentiation of granulosa cells into luteal-like cells (12, 13). However, research on human granulosa cells has primarily relied on cells obtained from patients undergoing in vitro fertilization (IVF), and cell lines such as; HGL5, HO-23, and HGP53, which have often been used as models to investigate the factors regulating granulosa cell proliferation and differentiation (14-16). However, these cells are typically obtained during luteinization due to the administration of human chorionic gonadotropin (hCG), which induces final maturation of the follicles and induction of ovulation and further initiating luteinization of the granulosa cells. This event decreases the expression of follicle-stimulating hormone receptors (FSHR), expressed on the granulosa cells, and excites the cell cycles (17-19). This pre-luteinized state limits use of these cells for studying early follicular development or the initial stages of luteinization.

The transition of granulosa cells from being responsive to follicle-stimulating hormone (FSH) to becoming more dependent on LH is initiated by a complex interaction of molecular and hormonal signals (20, 21). The change in steroidogenic profile during luteinization, as the expression of enzymes as 3*β*-hydroxysteroid dehydrogenase 2 (3*β*HSD2), steroidogenic acute regulatory protein (StAR), and cytochrome P450 family 11 subfamily A member 1 (CYP11A1), has been reported (5, 11, 19, 22, 23). However, the precise timeline and regulatory mechanisms governing this transition remain largely undefined, particularly in primary human granulosa cells rather than established cell lines.

Additionally, granulosa cells from different stages of follicular development express different phenotypic and functional characteristics. Granulosa cells from small antral follicles, which are in an earlier, less differentiated state, differ significantly from those obtained from preovulatory follicles in their steroidogenic profile, proliferation rates, and hormone responsiveness (24, 25). Understanding these differences is essential for developing reliable in vitro models of folliculogenesis and steroidogenesis, as well as for studying the molecular mechanisms regulating granulosa cell differentiation and luteinization in humans.

This study aims to address these critical gaps by providing a direct comparison of the luteinization timeline and steroidogenic changes in granulosa cells from small antral and preovulatory follicles in vitro. By investigating the intrinsic factors driving this transition—without external stimulation—our findings offer novel insights into the regulatory mechanisms of luteinization in humans. The goal is to determine the optimal experimental conditions and timeframes for examining granulosa cells prior to their differentiation into luteal cells. This knowledge could have significant implications for reproductive medicine, offering a foundation for developing new therapeutic strategies targeting infertility, ovarian dysfunction, and corpus luteum insufficiency.

## Methods

### Ethics

The collection of human samples and patient data from ovarian tissue through cryopreservation was approved by the Ministry of Health (J. no. 30-1372) and by the Danish authorities to comply with the European Union. The collection of patient material and data from women undergoing IVF treatment was approved by the Ministry of Health (H-23065318). Institutional Review Board (IRB) approval was obtained from Wake Forest University School of Medicine prior to obtaining granulosa-lutein cells as discarded biological material from patients undergoing IVF treatment (IRB#00084292).

Furthermore, written and oral informed consent were obtained from all participants prior to their inclusion in the study.

### Patient Populations

**Figure 1.**
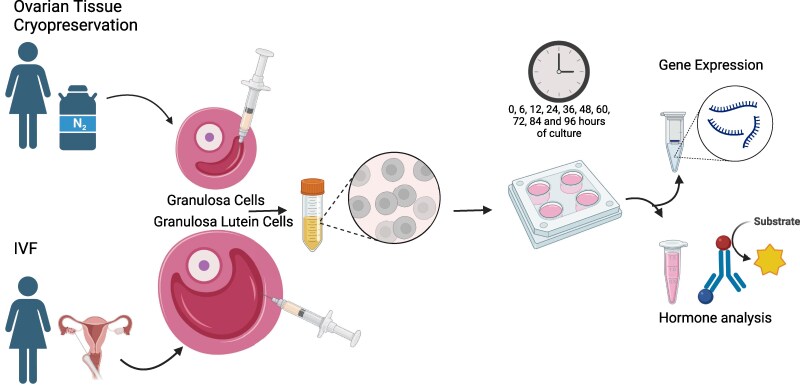
Experimental setup. Granulosa cells were obtained from small antral follicles donated by patients undergoing ovarian tissue cryopreservation, while granulosa-lutein cells were derived from preovulatory follicles donated by IVF patients. Follicular fluid containing granulosa cells was aspirated, followed by cell isolation. A total of 100 000 granulosa cells were seeded per well. Culture media were sampled for hormone analysis at 6, 12, 24, 36, 48, 60, 72, 84, and 96 hours. Granulosa and granulosa-lutein cells were collected for gene expression analysis at 0, 6, 12, 24, 36, 48, 60, 72, 84, and 96 hours.

Human granulosa-lutein cells were obtained from preovulatory follicles aspirated from patients undergoing IVF treatment at The Fertility Clinic, Department of Gynecology, Fertility and Obstetrics, at the University Hospital of Copenhagen (Fig. 1). A total of 12 patients donated granulosa-lutein cells from preovulatory follicles. Fertility treatment was initiated solely due to male factor infertility. Patient characteristics included women 31 to 40 years of age, serum anti-Müllerian hormone (AMH) level of 4.1 to 60 pmol/L, and a body mass index (BMI) ≥18.5 and ≤34. All IVF patients underwent ovarian stimulation using either a short antagonist or long agonist protocol with daily FSH doses ranging from 112.5 to 300 IU. Stimulation was performed with Menopur^®^ (hMG—G03GA02), and final oocyte maturation was triggered with Ovitrelle^®^ (choriongonadotropin alpha hCG—G03GA02) at a dose of 250 μg/mL, administered 36 hours before oocyte retrieval (Table 1).

**Table 1.** Patients’ characteristics, IVF treatment

IVF	P1	P2	P3	P4	P5	P6	P7	P8	P9	P10	P11	P12
**Age**	40	37	36	31	40	33	32	39	31	34	32	31
**BMI**	33.4	18.9	19.5	21.8	30.1	18.9	20.9	28	19.4	26.8	24.5	32
**AMH (pmol/L)**	7.3	14	8.6	13	4.1	25	23	22	10	60	6	27.9
**Stimulation Protocol**	Short	Short	Long	Long	Short	Short	Short	Short	Long	Short	Short	Short
**Menopur® (IU)**	225	300	225	300	300	225	150	112.5	225	300	300	137
**Ovitrelle® (ug/mL)**	250	250	250	250	250	250	250	250	250	250	250	250

Abbreviations: AMH, anti-Müllerian hormone; BMI, body mass index; IVF, in vitro fertilization.

Granulosa cells were obtained from small antral follicles of unstimulated ovaries from 18 women undergoing ovarian tissue cryopreservation for fertility preservation (Fig. 1). Patient characteristics included age 18 to 39 years, BMI <35 and serum AMH of >5.2 pmol/L. The diagnosis of the patients undergoing ovarian tissue cryopreservation for fertility preservation included breast cancer (n = 10), sarcoma (n = 5), and lymphoma (n = 3). The number of aspirated small antral follicles per patient was on average 5.7 ± 3.8 ranging from 4 to 9 follicles. Tissue from the corpus luteum was donated from 3 of the patients undergoing ovarian tissue cryopreservation. The ovarian tissue cryopreservation patients had no known endocrinological diseases, and all had normal AMH levels prior to surgery. As per the standard fertility preservation protocol, these individuals were scheduled to receive gonadotoxic treatments only after the ovarian tissue had been cryopreserved. Moreover, 5 patients undergoing IVF treatments agreed to donate follicle fluids to Wake Forest Institute for Regenerative Medicine, North Carolina, USA, to analyze the effect of testosterone and FSH on granulosa-lutein cells in vitro.

### Chemicals and Reagents

Culture media; *α*-MEM media (Gibco™ Life Tech. 22571-020), 1% Glutamax + (Gibco™, Life Tech. 35050-08), 0.01% Insulin-Transferrin-Selenium 100× (ThermoFisher 41400045), 5% Human Serum Albumin (CSL Behring, 109697, 20%), 1% Penicillin/Streptomycin (Invitrogen 15070-063). For treatment: 100 nM Testosterone (Sigma, T1500-5 g) and/or 25 ng/mL FSH (Rekovelle, Ferring), or Androstenedione (Merck, Cat: PHL-89562) alone was added to the culture media. For washing of the cells, phosphate buffered saline (PBS) (Gibco™, Life Tech., 1x) and HEPES buffer solution (Gibco, Cat: 15630049) was used.

Lymphoprep™ density gradient medium (StemCell Technologies, Cat: #07851) or Sigma Percoll gradient (Cat:#P4937). For RNA isolation; Trizol reagents (Ambion Life Technologies, 15596026), 1-bromo-3-chloropropane (Sigma, B9673-200ML), Ethanol 99%, DEPC H_2_O and RNeasy minikit 250 (Qiagen, 74106). RNA isolation of cDNA synthesis was performed using the high-capacity cDNA reverse Transcription Kit (Applied Biosystems™, 4368814).

Steroid content was measured with enzyme-linked immunosorbent assay (ELISA) assays, Progesterone (Demeditec Diagnostics Cat# DE1561, RRID:AB_3677532), Estradiol (Demeditec Diagnostics Cat# DE2693, RRID:AB_3677533) and Estradiol (Enzo Life Sciences Cat# ADI-900-008, RRID:AB_3677535).

### Granulosa Cell Isolation and Culture

Granulosa cells from small antral follicles and granulosa-lutein cells from IVF patients were isolated from pooled follicle fluids and cultured for up to 4 days.

After aspiration, during IVF or ovarian tissue cryopreservation, the follicle fluids were centrifuged at 400*g* for 5 to 10 minutes. The follicle fluid was discarded, and the pellet of cells was washed in prewarmed PBS. Figure 1 shows the experimental setup for this study focusing on the luteinization progression.

The granulosa cells, aspirated from small antral follicles, were resuspended in culture media after washing and plated in 4-well culture dishes (Nunc^®^) for incubation at 37 °C with 5% CO_2_ in a humidified incubator, as for the granulosa-lutein cells isolation.

Before culturing the granulosa-lutein cells aspirated in connection with oocyte pick-up from patients receiving IVF treatment were placed on a density gradient, and centrifuged at 400*g* at room temperature, with no brake (DEC: 1) for 30 minutes. The gradient medium formed a clear, transparent fluid at the top layer, while the erythrocytes had settled at the bottom of the tube. The granulosa-lutein cells were located at the interface of the gradient and were carefully aspirated using a Pasteur pipette and washed twice in prewarmed PBS. Cells were counted using cell Countess 3 (ThermoFisher Scientific) and seeded as 1 × 10^5^ cells per well in culture media. Culture conditions for granulosa cells obtained from small antral follicles and granulosa-lutein cells from preovulatory follicles were cultured for up to 96 hours, and samples taken every 12 hours of culture, from the control group and the group treated with androstenedione 100nM. For the long-term culture, granulosa-lutein cells from preovulatory follicles were cultured for 12 days, and samples taken every fourth day, for the control group treated with testosterone 100nM and treatment group supplemented with testosterone 100nM and FSH 25 ng/mL.

### Measurements of Progesterone and Estradiol Levels by ELISA

Progesterone (Demeditec, DE1561) and Estradiol (Demeditec, DE2693), (Enzo, ADI-901-008) levels were measured in the media obtained after granulosa cell culture, which was snap-frozen in liquid nitrogen until analysis. Culture media was diluted accordingly to an optimization run and analyzed, and controls and standards were made as described in the Demeditec or Enzo protocol for calculating the concentration of progesterone and estradiol in the culture media. Progesterone (Demeditec, DE1561) standard range 0.3 to 40 ng/mL, sensitivity 0.045 ng/mL, and for (17)estradiol (Demeditec, DE2693) standard range 25-2000 pg/mL, sensitivity 10.6 pg/mL. The intra-assay coefficient of variation (CV) ranged from 0.2% to 16.8% for (17)estradiol, and for progesterone CV ranged from 1.5% to 3.7%, while the inter-assay CV ranged from 5.3% to 11.3% for (17)estradiol, and from 0.7% to 8.1% for progesterone.

### RNA Isolation and Measurement

Granulosa cell samples stored in Trizol at −80 °C were thawed, vortexed, and incubated at room temperature before phase separation using 1-bromo-3-chloropropane. After centrifugation at 12 000*g*, the RNA-containing upper phase was collected and purified using the RNeasy silica membrane system. Ethanol and buffer washes were applied to remove contaminants, followed by elution with RNase-free water. The purified RNA was stored at −80 °C for further analysis.

RNA concentration and purity were assessed using a UV/Vis spectrophotometer (Beckman Coulter, Du 730). Absorbance at 260 nm was measured in duplicate to determine RNA concentration, which was then adjusted to 0.1 or 0.08 ug/μL before cDNA synthesis. Undiluted samples were stored at −80 °C.

### Gene Expression Analysis by Quantitative Reverse Transcription Polymerase Chain Reaction

From the granulosa cells from small antral follicles, cDNA was synthesized according to 80 ng RNA pr. sample, and from the granulosa-lutein cells cDNA was synthesized and normalized to 100 ng RNA pr. sample with the high-capacity cDNA reverse Transcription Kit (Applied Biosystems™, 4368814) based on the manufacturer's protocol and spin columns from Epoc Life Science (EconoSpin™ All-in-1 Mini Spin Columns, #1920) and then placed in the Arktik Thermal cycler (ThermoScientific, N11467). PCR for cDNA program started at 25 °C for primer annealing 10 minutes. Then 37 °C DNA polymerization at 120 minutes. 85 °C for 5 minutes. For enzyme deactivation and the last step until storage at a stable temperature 4 °C until storage at −20 °C.

Corpus luteum, tissue was incubated in RNAlater^®^-ICE at −80 °C for 12 hours, before RNA extraction was performed. The following genes were analyzed: Cytochrome P450 Family 19 Subfamily A Member 1 (*CYP19A1*), 17*β*-hydroxysteroid dehydrogenase 1 (*17βHSD1*), Steroidogenic acute regulatory protein (*STAR*), Luteinizing hormone choriogonadotropin receptor (*LHCGR*), Cytochrome P450 family 11 subfamily A member 1 (*CYP11A1*), Androgen Receptor (*AR*) and Marker of proliferation Kiel 67 (*MKI67*). Glyceraldehyde −3-phosphate dehydrogenase (*GAPDH*) served as the housekeeping gene (26). Expression of all the genes mentioned above were quantified using TaqMan assays from ThermoFisher Scientific, see Table 2. RT-PCR protocol used TaqMan Fast Advanced Master Mix (ThermoFisher, 4444557) applied with TaqMan probes in MicroAmp Optical 9-Well reaction plate (Applied Biosystems, N8010560) and run on a Quantstudio 3 Real-time PCR instrument and a comparative CT program. The comparative CT program is as following: preincubation 50 °C for 2 minutes, to 95 °C for 20 second, 1 cycle. Then amplification for 95 °C for 1 second, to 60 °C for 20 seconds, 40 cycles, and at last cooling at 60 °C for 20 seconds, 1 cycle. CT values below 38 were accepted. For the setup performed at Wake Forest Institute for Regenerative Medicine, North Carolina, USA, primers obtained from Integrated DNA Technologies (IDT), see Table 3, and PowerUp™ SYBR™ Green Master Mix (Applied Biosystems™, A25742) were used, and protocol setup was the same as for samples run with TaqMan probes. The data was then analyzed through QuantStudio™ Design & Analysis software (v1.5.2).

**Table 2.** TaqMan probes for RT-qPCR

TaqMan probe	Assay ID	Catalog no.
Human *HSD17β1*	Hs00166219_g1	4331182
Human *CYP19A1*	Hs00903411_m1	4331182
Human *STAR*	Hs00264912_m1	4331182
Human *CYP11A1*	Hs00167984_m1	4331182
Human *LHCGR*	Hs00174885_m1	4331182
Human *MKI67*	Hs04260396_g1	4448486
Human *AR*	Hs00171172_m1	4331182
Human *GAPDH*	Hs02786624_g1	4448486

**Table 3.** Primers SYBR green for RT-qPCR

Primers IDT SYBR GREEN	FWD Primer	REV Primer
*FSHR*	5′-TCA CCA AGC TTC GAG TCA TC-3′	5′-GGA GAA CAC ATC TGC CTC TAT C-3′
*CYP19A1*	5′-TGA CCA ATG AAT CGG GCT ATG-3′	5′-GTC CAA AGG GAT CCT CAA GAA G-3′
*GAPDH*	5′-ACC ACA GTC CAT GCC ATC AC-3′	5′-TCC ACC ACC CTG TTG CTG TA-3′

Abbreviation: RT-qPCR, quantitative reverse transcription polymerase chain reaction.

### Statistical Analysis

Statistical analysis was performed using R version 4.2.2 (27) (R Project for Statistical Computing (RRID:SCR_001905)) and the package “lme4” (28). Gene expression was analyzed by fitting a linear mixed model with patient as a random effect and androstenedione treatment and time as fixed effects. To evaluate the effect of time and androstenedione treatment on the concentration of (17)estradiol and progesterone, a linear regression model was fitted with concentration as an outcome and treatment and time as independent variables. Finally, the differences in gene expression between samples treated with FSH and control were analyzed by fitting an independent linear model per day with gene expression as an outcome and treatment as a variable. *P* values <.05 were considered significant.

## Results

### Granulosa Cell Culture Imaging

**Figure 2.**
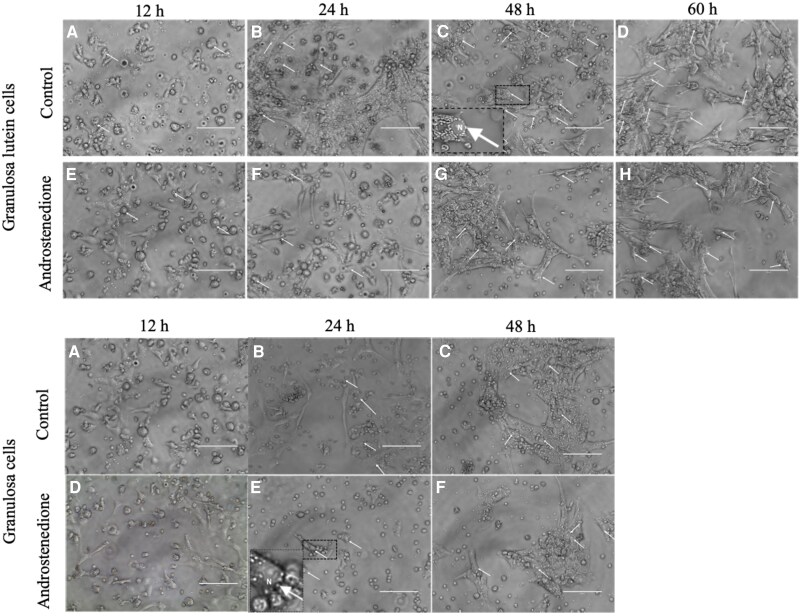
Lipid droplet accumulation in granulosa-lutein and granulosa cells over time. Granulosa-lutein cells isolated from preovulatory follicles, both control and androstenedione-treated, show comparable amounts of lipid droplets (indicated by white arrows). A slight increase in lipid droplet accumulation is observed at 48 hours compared to 12 and 24 hours. Similarly, granulosa cells isolated from small antral follicles, both control and androstenedione-treated, display a marked increase in lipid droplet accumulation after 48 hours of culture compared to earlier time points (12 and 24 hours). N = Nucleus. Images of cell culture (40X). Scale bar 100 μm.

Images of granulosa-lutein cells from preovulatory follicles and granulosa cells from small antral follicles were taken at different time points during culture with or without 100 nM androstenedione (Fig. 2). For both granulosa-lutein cells and granulosa cells from small antral follicles, the quantity of lipid droplets appears similar between control and androstenedione-treated groups. However, visually the lipid droplets seemed to be more prominent in granulosa-lutein cells than in granulosa cells from small antral follicles after 48 hours of culture.

### Relative Gene Expression of Luteinization, Granulosa Cell, and Proliferation Markers in Granulosa Cells From Small Antral and Granulosa-Lutein Cells From Preovulatory Follicles In Vitro

Tracking key gene expression markers for both undifferentiated granulosa cells and luteinized cells during culture is essential to determine whether they remain undifferentiated or become luteal cells. These markers can help pinpoint the timing of differentiation and luteinization. The baseline gene expression in both cell types corresponds to time 0 hours, without any culture period.

**Figure 3.**
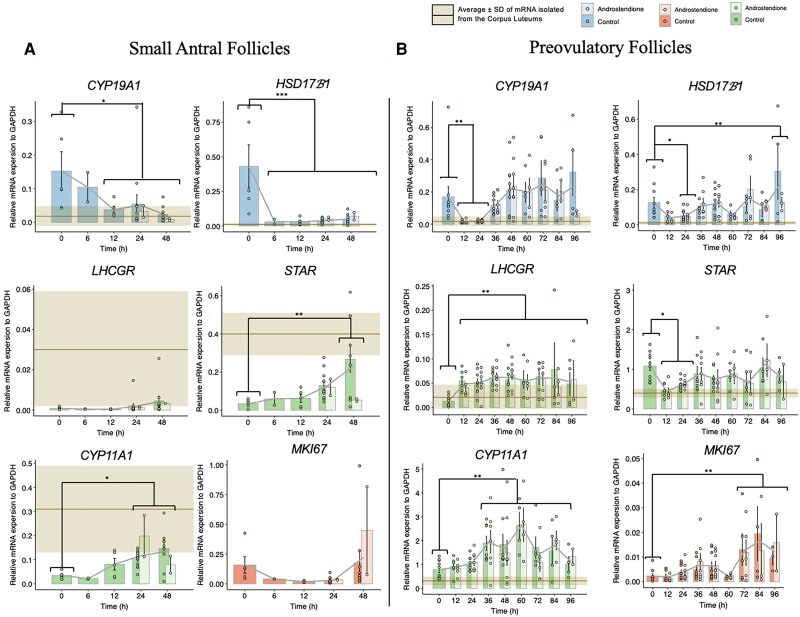
Gene expression (RT-qPCR). (A) Granulosa cells from small antral follicles, (B) granulosa-lutein cells from preovulatory follicles. Gene expression values for the corpus luteum are shown in yellow. No significant differences were observed between control and androstenedione-treated groups. Granulosa cells from small antral follicles initially exhibit high relative expression of *CYP19A1* and *HSD17β1*, key genes in estrogen production, with a decline observed after 6-12 hours. In contrast, *LHCGR*, *STAR*, and *CYP11A1* show an opposite trend, with increased expression after 24 to 48 hours. The relative mRNA expression of the proliferation marker *MKI67* is notably higher in granulosa cells from small antral follicles compared to granulosa-lutein cells from preovulatory follicles. Granulosa-lutein cells from preovulatory follicles display a decrease in *CYP19A1* and *HSD17β1* expression after 12 to 24 hours, followed by an increase in *HSD17β1* at 96 hours. *LHCGR* expression increases steadily from 12 to 96 hours, while *STAR* expression is initially high, decreases at 12 to 24 hours, and then stabilizes. *CYP11A1* expression increases after 36 hours and continues to rise until 96 hours. Significant *P* values are indicated as follows: *P ≤ .05, **P ≤ .01, ***P ≤ .001.

The relative gene expression of the granulosa cell markers: *CYP19A1* and *HSD17β1*, the luteinization markers: *LHCGR*, *STAR*, and *CYP11A1*, and the proliferation marker: *MKI67* was evaluated in granulosa cells from small antral follicles and granulosa-lutein cells from preovulatory follicles at different time points during culture with and without androstenedione (Fig. 3).

In small antral follicles, there were no statistically significant differences in the relative mRNA expression between the control and the cells supplemented with androstenediones across all studied genes (Fig. 3A). Since androstenedione treatment showed no effect, the statistical analysis reflects the overall impact of culture time on both control and androstenedione-treated cells.

There was a significant increase in the mRNA expression of the luteinization markers *STAR* from 0 to 48 hours (*STAR*: 0-48 hours *P* = .01 and *CYP11A1* from 0 hours to 24 hours and 48 hours (*CYP11A1*: 0-24 hours *P* = .017, 0-48 hours *P* = .005). Together with a significant decrease in gene expression of the granulosa cell marker for *CYP19A1* from 0 hours to 12 hours, 24 hours and 48 hours (*CYP19A1*: 0-12 hours *P* = .020, 0-24 hours *P* = .017, 0-48 hours *P* = .003) and for *HSD17β1* from 0 hours to 6 hours, 12 hours, 24 hours and 48 hours (*HSD17β1*: 0-6 hours, 12 hours, 24 hours and 48 hours *P* < .001) (Fig. 3A). There were no significant differences in the gene expression of *MKI67* in the small antral follicles.

**Figure 4.**
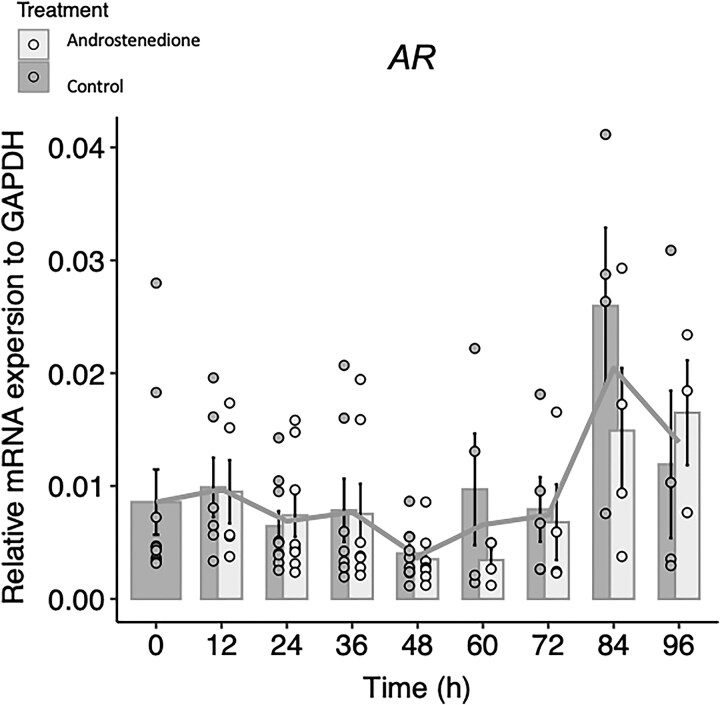
Gene expression (RT-qPCR). Expression of *AR* in granulosa-lutein cells from preovulatory follicles. No significant differences were observed between control and 100 nM androstenedione-treated groups at any time point. Significant *P* values are indicated as follows: *P ≤ .05, **P ≤ .01, ***P ≤ .001.

In the granulosa-lutein cells from preovulatory follicles, the relative mRNA expression of *CYP19A1*, *HSD17β1*, *LHCGR*, *STAR*, and *CYP11A1* and *MKI67* was not significantly different between the control and the androstenedione group across all the time points (Fig. 3B). Nevertheless, there was a significant decrease in mRNA expression of *CYP19A1* from time point 0 hours to time points 12 hours and 24 hours (0:12 hours, *P* = .043; 0:24 hours, *P* = .001) and of *HSD17β1* between the time point 0 hours and 24 hours and 96 hours (0:24 hours, *P* = .012; 0:96 hours, *P* = .001). Furthermore, for the luteinization markers, there was an increase in the gene expression of *LHCGR* from 0 hours to all the later time points (*P* < .01 all time points), there was also an increase in the expression of *CYP11A1* from time point 0 hours to 36 hours, 48 hours, 60 hours, 72 hours and 84 hours (0:36 hours, 48 hours, and 60 hours *P* < .01; 0:72 hours *P* = .009; 0:84 hours *P* = .006) and a decrease in gene expression of *STAR* from 0 hours to 12 hours and 24 hours (0:12, *P* = .006; 0-24 hours, *P* = .033). Finally, the expression of *MKI67* showed a significant increase between 0 hours and 72 hours, 84 hours, and 96 hours (0:72 hours, *P* = .001; 0:84 hours *P* > .001; 0:96 hours, *P* = .001) (Fig. 3B). To evaluate whether androstenedione treatment affected the expression of the androgen receptor (*AR*), relative mRNA levels were analyzed using quantitative reverse transcription polymerase chain reaction (RT-qPCR). As shown in Fig. 4, there was no significant difference in the relative mRNA expression of *AR* between the control group and the androstenedione-treated group.

### Concentration of (17)Estradiol and Progesterone Secreted by Granulosa Cells From Small Antral and Granulosa-Lutein Cells From Preovulatory Follicles In Vitro

**Figure 5.**
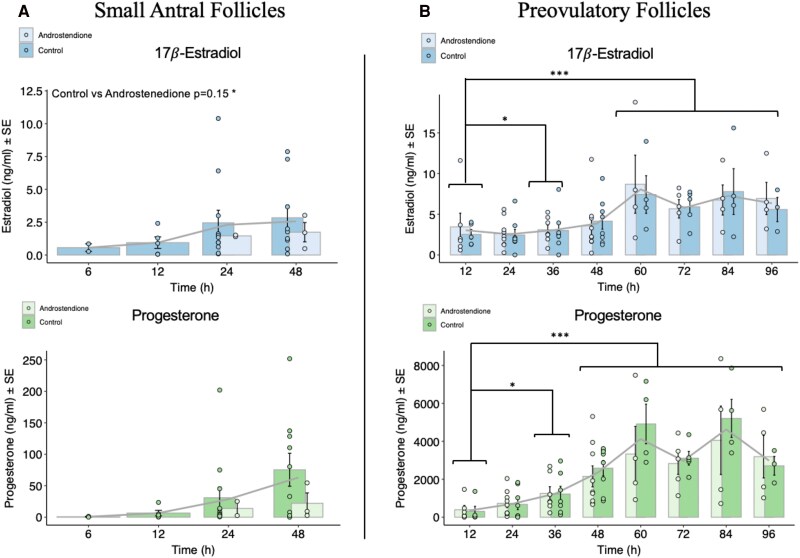
Hormone analysis (ELISA). (A) Media from the culture of granulosa cells from small antral follicles. (B) Media from the culture of granulosa-lutein cells from preovulatory follicles. No significant differences in 17*β*-Estradiol or progesterone production were observed between control and androstenedione-treated groups. (A) Granulosa cells from small antral follicles secrete increasing amounts of 17*β*-Estradiol and progesterone over time, although the increases were not statistically significant. (B) Granulosa-lutein cells from preovulatory follicles show a significant increase in 17*β*-Estradiol secretion after 36 hours, with an even greater increase from 60 to 96 hours. Progesterone secretion significantly increases after 36 hours, with further significant increases from 48 to 96 hours compared to time 0. Significant *P* values are indicated as follows: *P ≤ .05, **P ≤ .01, ***P ≤ .001.

The concentrations of estradiol and progesterone were measured in the spent culture media, collected every 12 hours from granulosa cells from small antral follicles and granulosa-lutein cells from preovulatory follicles (Fig. 5).

In granulosa cells aspirated from small antral follicles (Fig. 5A), there was a tendency towards a decrease, however, in the concentration of (17)estradiol due to the androstenedione treatment across time points (*P* = .15), while the concentration of progesterone was not altered by androstenedione supplementation (*P* = .212) (Fig. 5). When comparing the 6-hour time point with other time points, there were no significant differences either for (17)estradiol or progesterone (Fig. 5A).

In the granulosa-lutein cells obtained from the preovulatory follicles (Fig. 5B), the concentrations of (17)estradiol and progesterone secreted in vitro were not significantly different after treatment with androstenedione across the different time points. However, the concentration of (17)estradiol in the spent media was significantly higher at 36 hours, 60 hours, 72 hours, 84 hours, and 96 hours compared to 0 hours (0:36 hours, *P* = .031; 0:60 hours, 72 hours, 84 hours, and 96 hours, *P* < .001). Moreover, the concentration of progesterone in the spent media also significantly increased from 0 hours compared to 36 hours, 48 hours, 60 hours, 72 hours, 84 hours, and 96 hours (0:36 hours, *P* = .038; 0:48 hours, 60 hours, 72 hours, 84 hours, and 96 hours, *P* < .001) (Fig. 5B).

**Figure 6.**
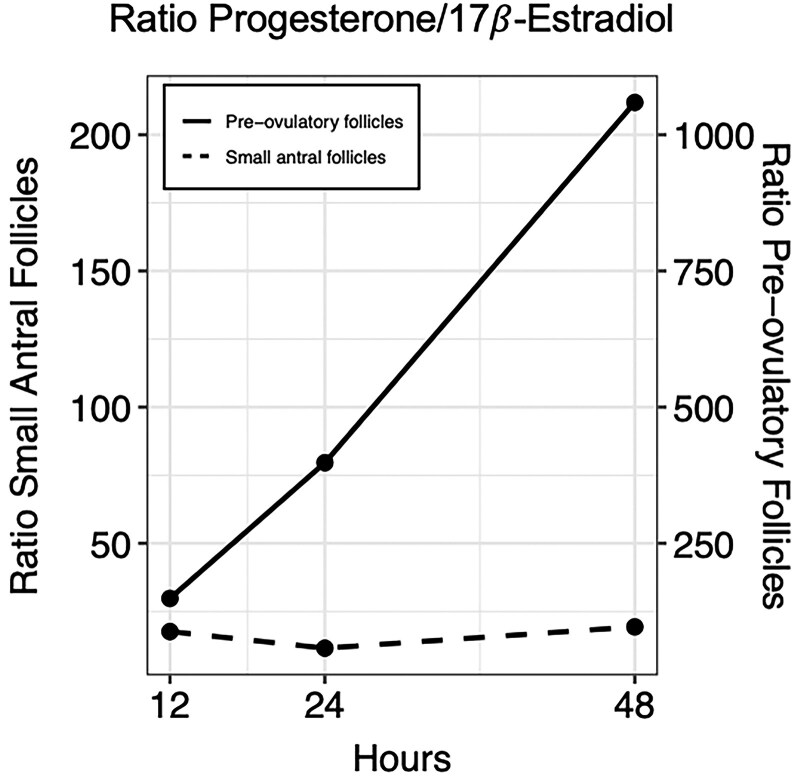
Ratio progesterone/17*β*-estradiol in vitro. The dashed line illustrated the progesterone/17*β*-Estradiol ratio in the spent media collected from granulosa cells isolated from small antral follicles through 48 hours of culture. The black line illustrates the progesterone/17*β*-Estradiol ratio in granulosa-lutein cells isolated from preovulatory follicles.

Comparing the medians of (17)estradiol secretion from granulosa cells from small antral follicles and granulosa-lutein cells from preovulatory follicles levels shows more than twice as high (17)estradiol production from the granulosa-lutein cells compared to granulosa cells after just 12 hours (Table 4). Moreover, comparing the medians of progesterone secretion from granulosa cells from small antral follicles and granulosa-lutein cells from preovulatory shows more than 82 times higher progesterone production in the granulosa-lutein cells compared to granulosa cells from small antral follicles after just 12 hours. After 48 hours the granulosa cells from small antral follicles begin to produce more progesterone, and the ratio decreases (Table 4). The progesterone to-(17)estradiol ratio in vitro exhibits distinct patterns depending on the origin of the granulosa cells. In the media from the granulosa-lutein cells isolated from preovulatory follicles, there is a rapid increase in progesterone production over 48 hours, leading to a marked rise in the ratio. In contrast, granulosa cells isolated from small antral follicles show a more balanced progesterone to-(17)estradiol ratio throughout the culture period, as illustrated in Fig. 6.

**Table 4.** Progesterone and 17*β*-estradiol median and interquartile range

17*β*-Estradiol (ng/mL)	Median	IQR	Progesterone (ng/mL)	Median	IQR
*Antral Follicles*			*Antral Follicles*		
6 h	0.6	2.9	*6* *h*	0.7	0.7
12 h	0.9	1.3	*12* *h*	3.3	4.7
24 h	1.4	2.4	*24 h*	4.9	12.3
48 h	1.9	3.7	*48* *h*	55.4	110
*Preovulatory Follicles*			*Preovulatory Follicles*		
12 h	2	2.5	*12* *h*	268	894
24 h	2.2	2.2	*24* *h*	939	1128
36 h	2.6	1.6	*36 h*	891	1459
48 h	2.8	2.8	*48* *h*	1648	2724
60 h	7.8	6.3	*60* *h*	2042	5050
72 h	6	2.2	*72* *h*	3075	1820
84 h	5.9	1.6	*84* *h*	1494	4260
96 h	5.6	3.2	*96* *h*	1599	3405

Abbreviation: IQR, interquartile range.

**Figure 7.**
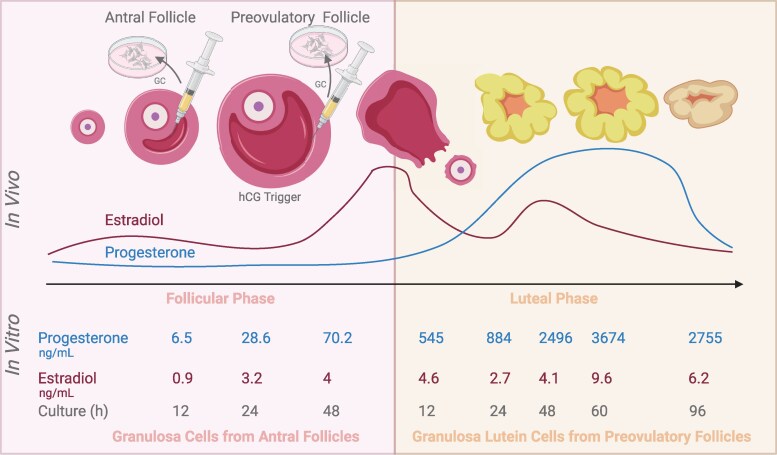
Progesterone and estradiol secretion patterns in vitro. Granulosa cells isolated from antral follicles during the follicular phase and granulosa-lutein cells isolated 36 hours post-hCG trigger during the luteal phase were cultured. Hormone secretion was measured in spent media. In the follicular phase (48-hour culture), estradiol and progesterone levels mimic in vivo patterns. In the luteal phase (96-hour culture), hormone fluctuations similarly reflect in vivo luteal phase dynamics, with distinct peaks of estradiol and progesterone.

The fluctuations in (17)estradiol and progesterone observed in the culture media correspond to the in vivo hormonal changes throughout the menstrual cycle. Granulosa cells isolated from small antral follicles closely resemble the in vivo hormonal profile of the follicular phase, characterized by balanced estradiol and progesterone levels. In contrast, granulosa-lutein cells isolated from preovulatory follicles mimic the luteal phase, with elevated progesterone secretion, as shown in Fig. 7.

### Relative Gene Expression of *FSHR*, *CYP19A1*, and (17)Estradiol Production in Granulosa-Lutein Cells From Preovulatory Follicles Treated With FSH and Testosterone In Vitro

**Figure 8.**
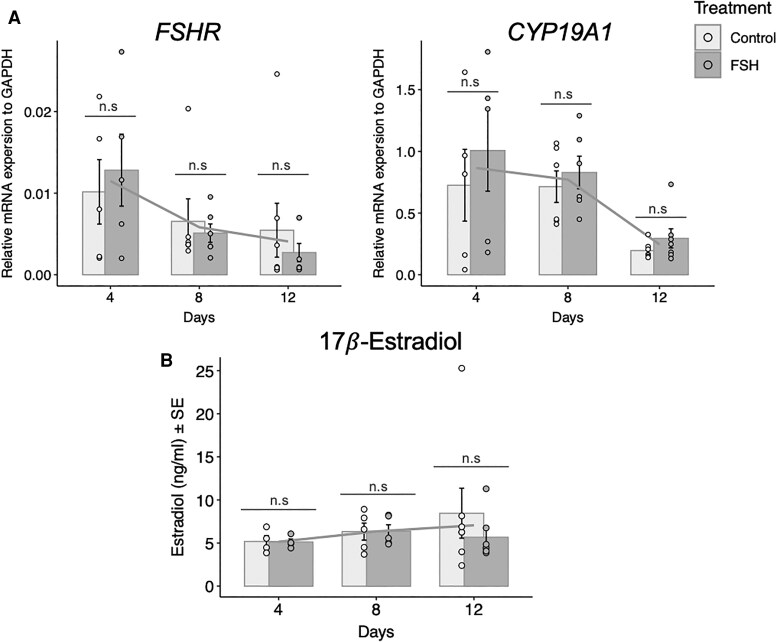
Gene expression and hormone analysis. (A) *FSHR* and *CYP19A1* gene expression in granulosa-lutein cells from preovulatory follicles. (B) 17*β*-Estradiol levels in spent culture media from granulosa-lutein cells treated as control (100 nM testosterone) or with 25 ng/mL FSH and 100nM testosterone. (A) No significant differences (n.s.) were observed in the mRNA expression of *FSHR* and *CYP19A1* between control and FSH-treated granulosa-lutein cells over 12 days. (B) 17*β*-Estradiol secretion showed no significant differences (n.s.) between control and FSH-treated groups at any of the 3 time points (days 4, 8, and 12). Significant *P* values are indicated as follows: *P ≤ .05, **P ≤ .01, ***P ≤ .001.

Granulosa-lutein cells were cultured for 12 days in the presence of 100nM testosterone (control) or in combination with 25 ng/mL FSH to study the long-term effect of culture on steroidogenesis. The relative mRNA expression of *CYP19A1* was not affected by any treatment. The relative mRNA expression of *FSHR* between control and FSH-treated groups expressed similar results, with no significant differences between treatment groups over time. On day 4, *P* = .665, day 8 *P* = .640, and day 12 *P* = .448 (Fig. 8A). As for the gene expression, the addition of testosterone alone or combined with FSH did not alter the secretion of (17)estradiol after 12 days of culture. Comparing control and treatment groups, day 4 *P* = .931, day 8 *P* = .968, and day 12 *P* = .381 (Fig. 8B).

## Discussion

This is the first study to characterize the luteinization profiles of human granulosa cells from both small antral and preovulatory follicles in vitro. Our finding showed distinct luteinization profiles when granulosa cells from these follicle stages were cultured. Granulosa cells from unstimulated small antral follicles were shown to be producing mostly estradiol and being in a proliferative state, but after 48 hours of culture, gene expression levels of luteinization markers resembled that of the corpus luteum. In contrast, granulosa-lutein cells from the hCG-triggered preovulatory follicles had already differentiated into lutein cells and produced high levels of both estradiol and progesterone. Hormone measurements showed an 82-times higher progesterone to estradiol ratio in the granulosa-lutein cells after 48 hours of culture compared to the follicular granulosa cells from the unstimulated small antral follicles.

Our results suggest that the granulosa-lutein cells are already highly luteinized before culture when aspirated from patients undergoing IVF treatment, and the upregulation of progesterone observed throughout the 96 hours may indicate a luteinization process of the remaining non-luteinized granulosa cells. These findings correlate with a study performed in marmosets, showing spontaneous luteinization of granulosa cells in culture (29). As their biological function, granulosa cells transform into luteal cells, and the dominant follicle will eventually become the corpus luteum gland (21, 30, 31). As a positive control, RNA was extracted from the corpus luteum of 3 patients undergoing ovarian tissue cryopreservation and analyzed for *CYP19A1*, *HSD17β1*, *CYP11A1*, *STAR*, and *MKI67*. The corpus luteum consists of small and large luteal cells derived from theca cells and granulosa cells, suggesting that the cells in the corpus luteum are fully luteinized (32). The granulosa-lutein cells from the preovulatory follicles exhibit gene expression levels for granulosa cell and luteinization markers that are higher than the relative mRNA expression observed, on average, in the corpus luteum. The increased expression of *LHCGR*, *STAR*, and *CYP11A1* compared to the corpus luteum suggests that granulosa-lutein cells from the aspirates are already highly luteinized. However, the simultaneous increase in granulosa cell markers, compared to the corpus luteum, indicates that these cells are not fully luteinized and continue to produce estradiol through the aromatase and FSHR pathway by converting estrone to estradiol. These findings fit the overall increase of estradiol in the media when comparing time points to the 12-hour time point, indicating an active estradiol production through the 96 hours. Comparing the regulation of estradiol and progesterone with the menstrual cycle, a similar pattern is seen in the concentrations measured in the culture media, a small peak in estradiol production and a crucial increase in progesterone levels in the luteinized granulosa cells (33, 34).

Granulosa cells from small antral follicles luteinize differently than granulosa-lutein cells from preovulatory follicles. Unlike preovulatory cells, they are not exposed to hCG before aspiration and remain in a proliferative state. In culture, CYP11A1 and STAR expression increase after 24 to 48 hours, while CYP19A1 and HSD17β1 decrease after 6 to 12 hours. Luteinization marker levels are initially lower than in the corpus luteum but reach similar levels by 48 hours, indicating incomplete luteinization. CYP19A1 and HSD17β1 expression significantly decline over time.

Estradiol and progesterone levels rise throughout the culture, reflecting in vivo hormonal fluctuations. The progesterone/estradiol ratio in granulosa-lutein cells is higher than in small antral follicle cells, indicating differentiation toward a luteal state. Secretion patterns, estradiol and progesterone levels increased throughout culture, mirroring in vivo fluctuations during the menstrual cycle (Fig. 7) (21, 35).

The ratio of progesterone/(17)estradiol in the culture of granulosa-lutein cells exceeds the ratio for the granulosa cells from small antral follicles, suggesting differentiation to a more luteal state.

When observing the morphological features of the cultured granulosa cells in our experiments, the luteinization process became visibly detectable by the presence of luteal lipid droplets in the granulosa cell cultures (36-38). The lipid droplets are small cholesterol storages, which contribute to the enormous progesterone production in the luteal cell, implying that granulosa cells reaching the luteal cell stage will produce more progesterone as they transition into cells with cholesterol storage. In the granulosa-lutein cells from the preovulatory stage follicle, the lipid droplets were observed immediately and increased over the duration of the culture. This is consistent with the significant high expression levels of *CYP11A1*, a key regulator of cholesterol conversion to progesterone (38), in granulosa-lutein cells after 36 hours and after 24 hours in the granulosa cells from the small antral follicles, since the lipid droplets in these cells are visible after 24 hours and highly noticeable after 48 hours. Nonetheless, since both types of granulosa cells spontaneously luteinize during culture, the preovulatory gonadotropin surge normally used to induce ovulation would not be the driving factor for these cells—especially not the granulosa cells from unstimulated small antral follicles. Animal studies have shown that both granulosa and theca cells spontaneously luteinize in vitro in serum-supplemented cultures (9, 11). Little is known about the mechanisms initiating and controlling luteinization, especially in human ovaries (11). Suggested factors include oocyte-specific proteins, the lack of androgen secretion from theca cells, intracellular signaling, and cell adhesion factors (11).

We hypothesized that supplementation with 100nM androstenedione or testosterone could potentially postpone luteinization in granulosa cells. However, no significant differences in gene expression or hormone secretion were observed in the granulosa-lutein cells aspirated from preovulatory follicles when androstenedione was added to the culture media. The theory behind this setup was that the additional high amount of androstenedione (100nM), biologically produced by the theca cells in the follicle, could accumulate a higher gene expression of *CYP19A1* in the granulosa cells and, hence increase the secretion of estrogens, prolonging a non-luteinized state of granulosa cells. Nonetheless, adding androstenedione did not result in an upregulation of either *CYP19A1* or secretion of estradiol in the culture media. In addition, androstenedione showed no effect on *HSD17β1*, *MKI67*, *STAR*, *CYP11A1*, or *LHCGR* expression compared to the control group. A similar result was obtained in the culture media analysis; no significant differences were observed in the progesterone production when adding androstenedione. This study further examined *AR* expression in granulosa-lutein cells, revealing no significant upregulation in *AR* expression in androstenedione-treated granulosa-lutein cells compared to the control group, suggesting no effect of exogenous androstenedione in the culture media.

The granulosa-lutein cells are aspirated from preovulatory follicles from patients undergoing fertility treatment who have been hCG triggered. This may have accelerated luteinization of the granulosa cells before aspiration, which occurs 36 hours after the hCG trigger, suggesting a transition to androgen independence from the theca cells. Consequently, this shift could account for the absence of any effects to exogenous androstenedione. Another issue could be the lack of FSH in the culture media, as FSH binds to the FSH receptor on the cell surface of granulosa cells and thereby activates a cascade of intracellular events primarily led by cyclic AMP (cAMP) for upregulation of the *CYP19A1* expression and enhances aromatase activity, which will increase estradiol production (39-41). The addition of FSH may facilitate the conversion of exogenous androstenedione to estradiol. A study found that FSH enhances aromatase activity in cultured human cumulus cells (42). Similarly, Sjögren et al studied granulosa-lutein cells from preovulatory follicles, showing no estradiol increase with FSH or LH alone. However, adding 300 nM testosterone led to a 50- to 100-fold rise in estradiol production (20).

Our study on the long-term culture of granulosa-lutein cells aspirated from preovulatory follicles revealed no upregulation of (17)estradiol secretion over 12 days of culture, even with the addition of 25 ng/mL FSH and 100nM testosterone. Furthermore, no significant differences in the relative mRNA expression of *FSHR* and *CYP19A1* were found between the control group (treated with testosterone alone) and the treatment group (treated with both FSH and testosterone). These findings suggest that granulosa-lutein cells, once aspirated at the onset of luteinization, are unable to revert to an undifferentiated granulosa cell phenotype characterized by *FSHR* expression and primary estradiol production.

Our study shows a significant increase in *MKI67* expression in granulosa-lutein cells after 72 hours. However, compared to granulosa cells from small antral follicles, *MKI67* levels are notably lower. Small antral follicle cells exhibit a 10-fold higher expression, indicating greater proliferative potential. This reduced *MKI67* expression in granulosa-lutein cells resembles the pattern seen in bovine luteal cells during luteolysis, where cells reenter the cycle before apoptosis (31). This suggests that the next step for our granulosa-lutein cells could also be luteolysis.

Clinically, these findings have significant implications for assisted reproductive technologies (ART). Premature luteinization of granulosa cells can negatively impact oocyte quality and embryo development, affecting IVF success rates (43-45). A better understanding of this process can help refine ovarian stimulation protocols to optimize ART outcomes. Furthermore, given the role of luteinized granulosa cells in progesterone secretion, these insights contribute to improving luteal phase support strategies in ART and addressing luteal phase deficiencies in both natural and stimulated cycles.

The limitations of this study include the limited availability of granulosa cells from small antral follicles collected from women undergoing ovarian tissue cryopreservation. Additionally, we acknowledge that complete patient characteristics for all ovarian tissue cryopreservation patients were unavailable, as samples were collected from different hospitals. However, comprehensive clinical data were available for IVF patients treated at the University Hospital of Copenhagen. Furthermore, experimental studies with androgen and FSH supplementation were conducted only in granulosa-lutein cells from preovulatory IVF follicles, using 2 different androgens. This variation may limit direct comparisons between long-term and short-term exposure effects. Further studies are needed to clarify the role of FSH and androgen signaling in small antral follicles during luteinization.

In conclusion, this study demonstrates that granulosa cells from small antral follicles and preovulatory follicles undergo spontaneous luteinization in vitro, with distinct timelines and responses to culture conditions. Granulosa-lutein cells are already highly luteinized at the time of aspiration and cannot revert to a pre-luteinized phenotype, while granulosa cells from small antral follicles gradually transition into luteal cells. These findings suggest that luteinization in vitro is driven by intrinsic cellular changes rather than external triggers and could potentially be influenced by the absence of oocyte-specific signals and theca cells in culture. These findings enhance our understanding of follicular development and luteinization, providing a foundation for the development of new therapeutic strategies for reproductive disorders and infertility.

## Data Availability

Some or all datasets generated during and/or analyzed during the current study are not publicly available but are available from the corresponding author on reasonable request.

## Abbreviations

AMH: anti-Müllerian hormone
AR: androgen receptor
BMI: body mass index
CYP11A1: cytochrome P450 family 11 subfamily A member 1
ELISA: enzyme-linked immunosorbent assay
FSH: follicle-stimulating hormone
FSHR: follicle-stimulating hormone receptor
hCG: human chorionic gonadotropin
IVF: in vitro fertilization
LH: luteinizing hormone
LHCGR: luteinizing hormone/choriogonadotropin receptor
PBS: phosphate-buffered saline
RT-qPCR: quantitative reverse transcription polymerase chain reaction
StAR: steroidogenic acute regulatory protein
